# Crude Blunders

**Published:** 1881-09

**Authors:** 


*A little more thoughtfulness* and study would save us sometimes from crude blunders. Not long ago a dentist reported the cure of a polypus of the antrum by the injection, *through the nostril*, of a solution of nitrate of silver, repeated a few times! It is some comfort to know that he was an "M. D." at that; and we suppose his diploma would give him place among the "doctors" proper, in spite of this remarkable cure. Well, it is a comfort at least to know that dentistry will not be blamed this time. H.

